# Thermal Decomposition Behavior of Polyimide Containing Flame Retardant SiO_2_ and Mg(OH)_2_

**DOI:** 10.3390/polym14142791

**Published:** 2022-07-08

**Authors:** Jie Wang, Aifeng Jiang, Yanchun Li, Dongming Song, Yifan Li, Long Cheng

**Affiliations:** 1 School of Chemistry and Chemical Engineering, Nanjing University of Science and Technology, Nanjing 210094, China; jiewang@njust.edu.cn (J.W.); songdm@njust.edu.cn (D.S.); yifanmaxlee@njust.edu.cn (Y.L.); lc@163.com (L.C.); 2 Southwest Technology and Engineering Research Institute, Chongqing 400039, China

**Keywords:** polyimide, degradation, thermodynamics, aging

## Abstract

The effects of flame retardant silica (SiO_2_) and magnesium hydroxide (Mg(OH)_2_) on the thermal decomposition process of polyimide (PI) are discussed in this paper. Firstly, the decomposition process of PI in a nitrogen and oxygen atmosphere was studied by thermogravimetric analysis and differential scanning calorimetry methods, and the kinetic parameters were calculated by the nonlinear fitting method. In an inert atmosphere, PI decomposition consists of a three-step endothermic reaction, whereas in an oxygen atmosphere, PI decomposition consists of two steps, in which the first step does not change, and the second step changes to a violent exothermic peak. The effects of 3 wt % SiO_2_ (SPI) and 3 wt % Mg(OH)_2_ (MPI) on the degradation kinetics of PI are discussed. The results show that under an oxygen atmosphere, SiO_2_ and Mg(OH)_2_ hydroxide mainly delayed the second-step oxidation exothermic peak temperature of PI by 5 °C. In summary, the first step of the PI degradation is not affected by oxygen, and the flame retardant mainly acts in the second step, which can delay the oxidation heat release. In addition, the addition of SiO_2_ could prevent PI from aging whereas Mg(OH)_2_ has barely effect on the aging of PI.

## 1. Introduction

With the increase in the awareness of fire accidents, it is desirable to find a material that can be applied in a wide variety of high-temperature environments. Polyimide has attracted attention from researchers due to its excellent strength, outstanding thermal stability, and high thermal insulation. It has been widely used in many fields, such as aerospace, electronic technology, and new energy [1,2,3,4,5]. Since 1961, when the first polyimide film was produced by Dupont, polyimide has been considered an ideal material in the industrial filed [6]. Several products of polyimide, such as foams, coatings, adhesives, and fibers, etc., were applied successively, implying that the development of polyimide has a potential applicability in many fields [7,8]. Thereafter, polyether amide materials, Usilex R polyimide films, thermoplastic polyimide, etc., have been developed by the USA, Japan, and other countries [9,10]. Certainly, the development of polyimide still continues in order to meet the high demand throughout the world for polyimide.

Due to its excellent properties, a series of polyimide with inorganic materials also have prospects within flame-retardant fields [11,12,13]. The inorganic additives, such as silica, graphene, hydroxides, etc., are used to modify polyimide, and the composites are usually made into films, foams, and aerogels [14,15]. Mg(OH)_2_ has a high decomposition temperature (340–490 °C) and a low heat absorption, whose performance in inhibiting the temperature rise in a material is worse than that of Al(OH)_3_, whereas its carbonization and flame-retardant effect on polymers is better than that of Al(OH)_3_. Wang [16] prepared nano-Mg(OH)_2_/PI composite films with varying contents of Mg(OH)_2_ by in situ polymerization and thermal imidization successfully. The results of the thermal analysis indicate that the thermal stability of the nano-Mg(OH)_2_/PI composite films decreased with the increasing of Mg(OH)_2_ compared with pure PI, whereas the Young’s modulus of PI-3% was 2851.6 N/mm^2^ and increased by 155% relative to that of pure PI.

Nano-SiO_2_ has the advantages of low density, high strength, high toughness, and excellent stability at high temperature, etc., and is mostly used in the modification of polymers [17]. A porous nano-polyimide/silica (PI/SiO_2_) aerogel with excellent fire resistance was prepared via the co-gel method, which has a low flame propagation rate between 25 and 300 °C (0.2–0.6 W·s^−1^) and a low thermal conductivity (31.1–58.5 mW·m^−1^·K^−1^) [18]. So far, research on PI with SiO_2_ or Mg(OH)_2_ composites has mainly focused on its application in fire resistance, whereas only a few studies have addressed the decomposition mechanism function of polyimide in the flame-retardant field.

The carbonization of polyimide “Kapton” and “Novax” film has been studied [19,20]. The results show that the decomposition process of polyimide “Kapton” is a two-step reaction. Firstly, O_2_ is released, and the crystalline polyimide is transformed into an amorphous form. The escape of nitrogen and the remaining oxygen occurs in the second step. Meanwhile, hexagonal carbon layers are observed. The decomposition process of polyimide “Novax” is also a two-step reaction. CO and CO_2_ are released in the first step (450–600 °C) with obvious weightlessness. In the second process, the weight loss is mainly caused by the generation of N_2_ above 800 °C. Zhao [21] also reported the pyrolysis of polyimide “Kapton” film. From the results, we can see that the content of carbon increased sharply at 550–700 °C, which is ascribed to the thermal condensation of the molecules resulting in the cleavage of the C-O, C-N bonds to form C-C, C=N bonds. The increase in oxygen is likely the result of the active sites on the sample surface reacting with the oxygen in the air above 800 °C. The breakage of the C-N bond produces N_2_ and HCN, which results in the decrease in the ratio of nitrogen. In these works, the focus of the analytical methods is on X-ray diffraction (XRD), thermogravimetric analysis (TGA), gas phase monitoring methods, etc., whereas the DSC method is not mentioned. 

From the abovementioned literature, the work showed that the pyrolysis of polyimide is so complex that conventional dynamic methods are not suitable for its decomposition processes. Hence, a multivariate nonlinear fitting method was proposed to investigate the mode of action of PI. The multivariate nonlinear fitting method, basing on the iso-conversional method, fit the results of the thermogravimetric analysis (TGA) curves with the Gauss–Newton method [22,23]. The best dynamic results of each step were compared to obtain the kinetic parameters. 

Herein, the decomposition process of PI was examined by the TG–DSC method, and the multivariate nonlinear fitting method was used to determine the kinetic parameters of each step. To study the influences of the decomposition process and mode of action on PI containing different inorganic modifiers, SiO_2_ and Mg(OH)_2_ were selected to modify PI. The decomposition process and mode of action of PI containing SiO_2_ and Mg(OH)_2_ were obtained. The simulated and experimental data on the aging of the three substances were both examined at 500 °C for 24 h to confirm the kinetic parameters and reaction models. This work may provide a theoretical basis for the decomposition and kinetic behaviors of polyimide at high temperature.

## 2. Experimental

### 2.1. Materials

Experimental materials are presented in Table 1.

### 2.2. Preparation of Silica/Polyimide (SPI), Magnesium Hydroxide/Polyimide (MPI) 

Polyimide was mechanically agitated for 30 min, weighed, and put into an oven for use. According to the Ref. [24], 3 wt % of SiO_2_ was stirred with polyimide for 30 min, and put into an oven for use; then, SPI (SiO_2_/PI) was obtained. The preparation of MPI (Mg(OH)_2_/PI) was the same as for SPI.

### 2.3. Characterization

Thermogravimetric analysis (TGA) and differential scanning calorimetry (DSC) was carried out on a TGA/DSC 3+ (METTLER TOLEDO, Shanghai) under nitrogen and oxygen atmospheres with a flow rate of 20 mL·min^−1^. The temperature ranged from 30 to 1000 °C in an alumina crucible with a 1 mm hole in the lid and a volume of 70 μL, and the sample mass ranged from 1 to 2 mg with heating rates of 20, 15, 10, and 5 K·min^−^^1^. 

NETZSCH Thermokinetics 3.0, which is a software for the kinetic evaluation of thermal measurements performed, was used to calculate the activation energy of PI, SPI, and MPI. Access to the data from different instruments is possible via the general interface ASCII file. All types of data acquired using Thermogravimetry (TG), differential thermal analysis (DTA), differential scanning calorimetry (DSC) or mass spectrometry (MS) can be evaluated. The software is based on the Friedman or Ozawa–Flynn–Wall analysis, or ASTM E698 analysis to obtain the activation energy. 

## 3. Results and Discussion

### 3.1. Thermal Stability of Polyimide 

**Figure 1:**
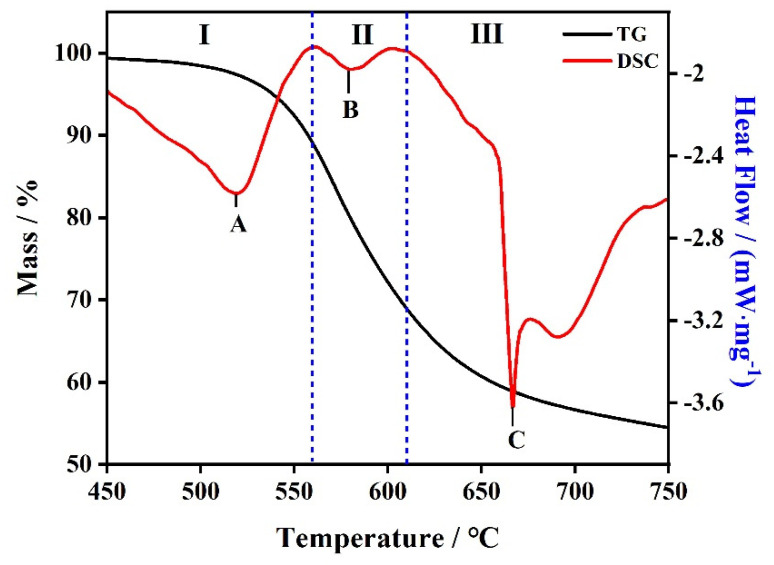
TG−DSC curves of PI under an N_2_ atmosphere.

TG–DSC curves are applied to analyze the thermal stability of PI from 450 to 750 °C with a heating rate of 20 K·min^−1^ under an N_2_ atmosphere. As shown in Figure 1, the onset temperature, the inflection temperature, and the final temperature are 538 °C, 572 °C, and 627 °C, respectively. The total weight loss of polyimide is 45.2%. Therefore, we infer that the decomposition process of polyimide is a one-stage reaction.

**Figure 2:**
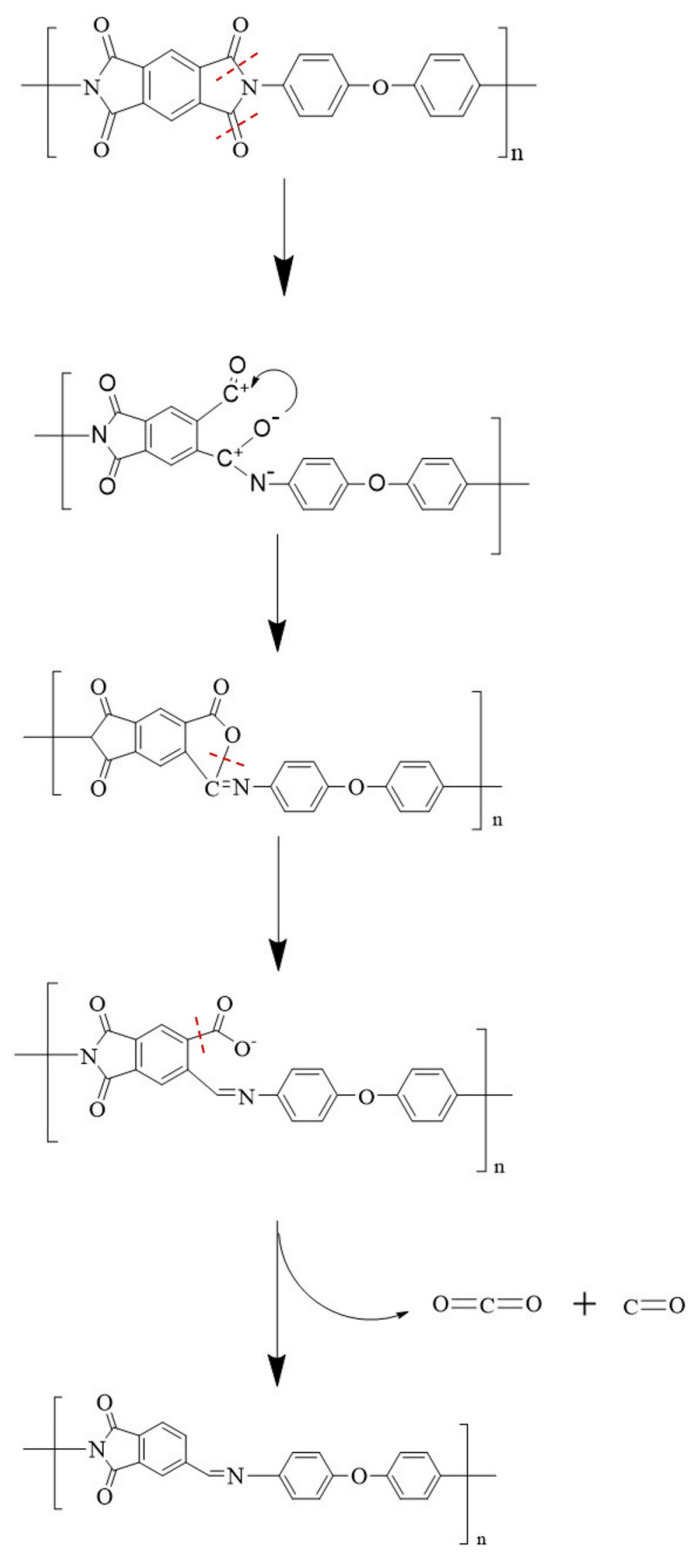
A scheme reporting the chemical decomposition of PI.

The DSC curves of the decomposition of polyimide have not been mentioned in many studies [25,26,27]; so, the mode of action of polyimide was further discussed (the red curve in Figure 1). There are three endothermic peaks of the DSC curve, which are peak A (P_A_ 520 °C), peak B (P_B_ 580 °C), and peak C (P_C_ 667 °C), respectively. P_A_ is the decomposition endothermic peak of polyimide, which could be ascribed to the breaking of the C=O group in the amide ring of PI, to generating part of CO and CO_2_ [19]. CO_2_ is produced by the residues of the intermediate raw materials before imidization. The heat absorption of P_B_ is weak, which causes the fracture of the remaining C=O bands in the imine ring to release CO and CO_2_. The results of the generation of CO of P_A_ and P_B_ indicate that the C=O group is not broken simultaneously. P_C_ is generated by the cleavage of the remaining oxygen-nitrogen functional groups to form a nitrogenous substance and nitrogen-oxide [19]. A scheme reporting the chemical decomposition of PI is shown in Figure 2. According to the DSC curve, the pyrolysis reactions of PI could be judged a three-step endothermic reaction.

**Figure 3:**
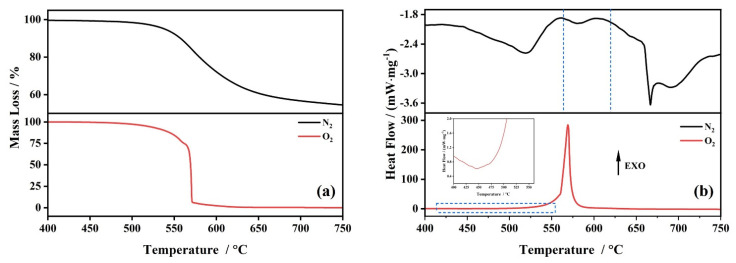
TG (**a**) and DSC (**b**) curves of PI under N_2_ and O_2_ atmospheres.

The mass loss of PI is nearly 100% under an O_2_ atmosphere, as shown in Figure 3a. The onset temperature was 568 °C, which increased by 30 °C, compared to the N_2_ atmosphere, whereas the reaction is almost complete when the temperature is about 571 °C. The results reveal that the product by the decomposition reaction of PI under an O_2_ atmosphere led to the rate of decomposition and the mass loss increasing.

The DSC curve (the red one) implied that the decomposition of PI is an exothermic reaction in an O_2_ atmosphere with a peak temperature of 569 °C, as shown in Figure 3b. There is also an endothermic peak between 420 and 480 °C. Compared to the DSC curve of PI in an N_2_ atmosphere (the black one), this endothermic peak was the first decomposition of PI, whereas the second one changes from endothermic to exothermic, which suggests that O_2_ reacted with CO generated by the decomposition of PI and released a large amount of heat. The results reveal that the first decomposition of PI is irrelevant to the ambient of PI.

### 3.2. The Influence of SiO_2_ and Mg(OH)_2_ on the Thermal Stability of Polyimide 

**Figure 4:**
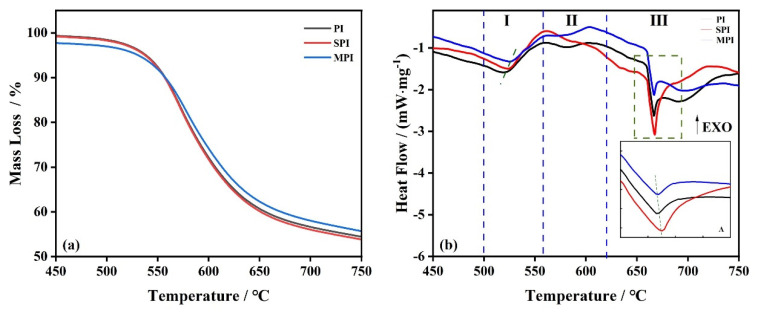
TG (**a**) and DSC (**b**) curves of three substances under an N_2_ atmosphere.

To analyze the influence of the additives of SiO_2_ and Mg(OH)_2_ on the decomposition of PI, the samples of SPI and MPI are studied at the same experimental conditions, respectively. The results are shown in Figure 4a TG curves, and Figure 4b DSC curves. In Figure 4a, the mass loss of SPI is similar to PI. For the sample of MPI (the blue curve), 2% weight loss occurs at 359 °C due to the low decomposition temperature of Mg(OH)_2_, which leads to the starting point not being at 100% in Figure 4a, and also causes the decomposition temperature of MPI to be earlier than for PI. 

As Figure 4b depicts, the three peaks of PI all shift after mixing with SiO_2_ and Mg(OH)_2_. The experimental data are shown in Table 2. For SPI, the shift of the P_A_ to the right is accompanied with a decrease in the heat, whereas there is little change in the P_B_. The results illustrate that the three-dimensional network structure of SiO_2_ inhibited the decomposition of PI and hindered the transfer of heat [28]. For MPI, the shift of the P_A_ and P_B_ to the right is ascribed to MgO, produced by the decomposition of Mg(OH)_2_, and covering the surface of PI, which prevents the decomposition of PI and causes the P_A_ and P_B_ to move towards a high temperature. The P_C_ of SiO_2_ and Mg(OH)_2_ have the same shift. However, the mass loss of the third stage of SPI is less than for PI, which is attributed to the different hydroxyl groups on the surface of SiO_2_, which can catch the radicals generated by PI to inhibit the decomposition of PI.

**Figure 5:**
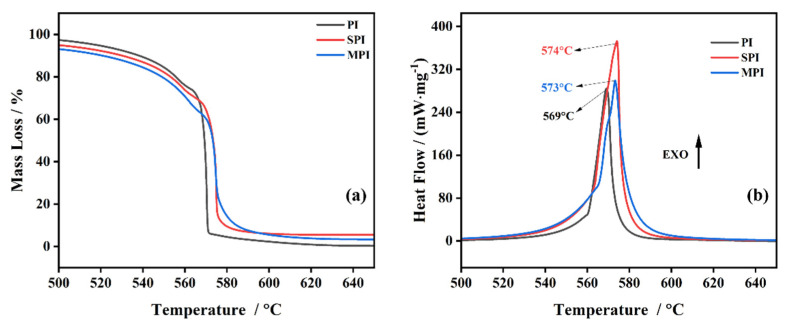
TG (**a**) and DSC (**b**) curves of the three substances under an O_2_ atmosphere.

The TG curves of PI, SPI, and MPI are shown in Figure 5a with a heating rate of 20 K·min^−1^ under an O_2_ atmosphere. The onset temperature and the final temperature of PI are 568 °C and 571 °C, respectively. The mass loss approaches 100%. The decomposition temperature of SPI is 571 °C, and at the end temperature of 575 °C, the weight loss was 92%; MPI starts to decompose at 570 °C, and the reaction is complete at 574 °C, with a mass loss of 93%.

The peak temperatures of the DSC curves of PI, SPI, and MPI are 569 °C, 573 °C, and 574 °C, respectively. The results imply that the additions of SiO_2_ and Mg(OH)_2_ have little effect on the first decomposition of PI under an O_2_ atmosphere, whereas the peak temperatures of the second step of SPI and MPI increased by 5 °C compared with that of PI.

### 3.3. Kinetic Analysis of Polyimide

#### 3.3.1. Calculation of Activation Energy

**Figure 6:**
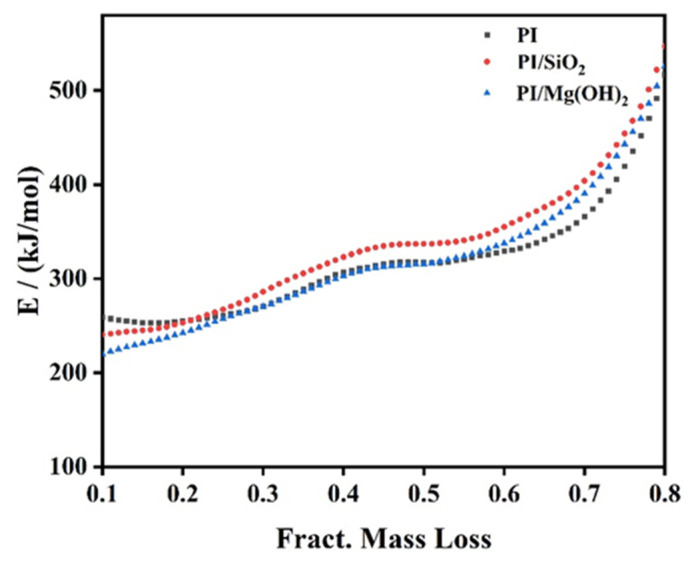
Results of the Friedman calculation.

The Friedman method is the most widely used differential isoconversional methods for kinetic analysis [29], which is the model-free (isoconversional) method of kinetic analysis calculating dependence of activation energy E(α) on degree of conversion α. As Figure 6 depicts, the model-free Friedman method is employed to calculate the activation energy (E) of PI, SPI, and MPI to confirm the mode of action. For SPI, the E value is lower than that of PI when the mass loss is less than 21%. The results indicate that it is easier to lead to weight loss of SPI than PI, which is consistent with the changes in the weight loss of PI and SPI in the first stage. When the mass loss is higher than 21%, the E value of SPI is always greater than that of PI. The results elucidate that the pyrolysis of SPI is difficult, and the conversion rate decreased, which is in accord with the changes in weight in the second and third steps in Table 1, as the three-dimensional network structure of SiO_2_ is beneficial to the formation of a continuous and dense carbon layer to prevent the pyrolysis of PI. The change in the E value of SPI shows that its decomposition is at least a two-step reaction.

Compared to PI, the E value of MPI could be divided into three stages based on the change in the mass loss. When the mass loss is less than 27%, the E value is less than that of PI; the reason why the E value decreases is that the low melting point of Mg(OH)_2_ made it decompose in the early stage. The E value is close to that of PI between 27% and 55%, indicating that the H_2_O produced by the pyrolysis of Mg(OH)_2_ could absorb the heat, which slows down the decomposition of PI. When the mass loss is higher than 55%, the E value is greater than that of PI. The results show that MgO generated by the decomposition of Mg(OH)_2_ covers the surface of PI, isolating PI from the O_2_, which makes the decomposition reactions of PI difficult and increases the E value of MPI. The changes in the E value of MPI suggests that the decomposition process of MPI is at least a three-step reaction. 

For elementary reactions, the E value is constant with conversion or temperature, whereas the E value of most reactions is the sum of the complex processes, so the change in the activation energy with conversion means that the sum of the activation energies of multiple reactions have changed. After adding flame retardant, activation energy change means that mode of action changed. This will be further explained in the following part Section 3.3.2.

#### 3.3.2. Multivariate Nonlinear Fitting

**Figure 7:**
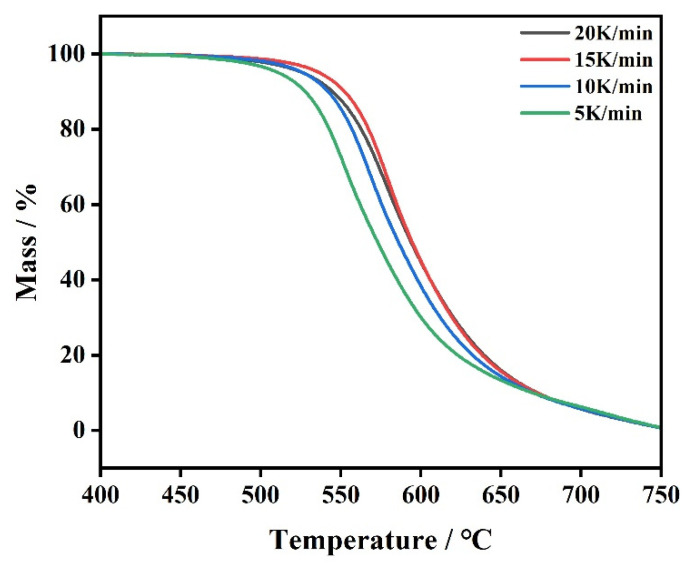
Multivariate nonlinear fitting results of polyimide.

From the abovementioned, although there is only one-stage loss weight of the TG curve of PI, the decomposition process is complicated; so, the conventional dynamic models could not describe the mechanism of PI effectively. Therefore, we used the multivariate nonlinear fitting method to analyze the pyrolysis of PI and selected the decomposition model of A-1→B-2→C-3→D to fit. The fitting curves are displayed in Figure 7, and the kinetic parameters are listed in Table 3.

The fitting results indicates that the decomposition processes of the three substances are probably three steps, which is consistent with the results of the thermal analysis. The decomposition mechanism of PI may be A_2_ + A_n_ + C_1_, which represent that the reaction model of PI is the A_2_ model—2-dim. Avrami-Erofeev in the first step and the one of the second-step is A_n_ model—n-dim. Avrami-Erofeev, indicating that the models of the first and the second steps of PI are random nucleation and a subsequent growth model. Similarly, the A_2_ model is appropriate for the mechanism of SPI and MPI in the first step, and the A_n_ model is suitable for the mechanism of SPI and MPI in the second step. The F_n_ model met the third-step mechanism of SPI, and the F_2_ model is appropriate for that of MPI. 

The results of fitting are listed in Table 3. Apparently, the first-step E value of PI is higher than those of SPI and MPI, the second one is close to that of MPI but less than the one of SPI, and the E value of the third is less than those of SPI and MPI. The results of the optimum are consistent with the calculation using the Friedman method. 

#### 3.3.3. Lifetime Prediction of Three Substances

**Figure 8:**
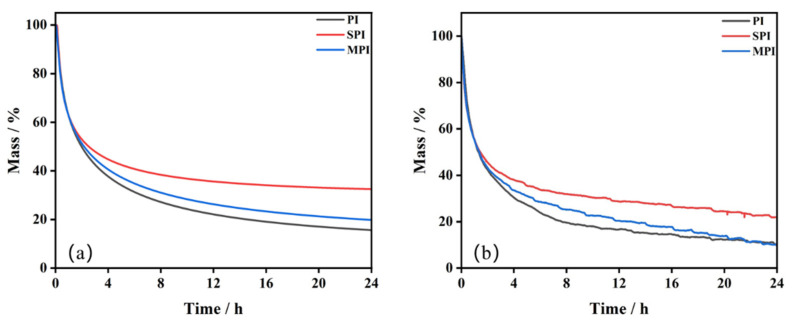
Mass loss of the three substances at 500 °C for 24 h: (**a**) prediction; (**b**) experiment.

Based on the results of the abovementioned multiple nonlinear fitting, the NETZSCH Thermokinetics 3.0 software is used to predict the aging of the three substances at 500 °C for 24 h. The results are shown in Figure 8a. From the results of the prediction, we can see that the decomposition process of the three substances is similar. The conversions of PI, SPI, and MPI are 84.4%, 67.5% and 80.2%, respectively.

In order to verify the validity of the reaction mechanism functions, the thermal aging of the three substances is examined at the same conditions. The results are shown in Figure 8b. The overall trend of the curves matches with the predictions, but the conversions are different (89.8%, 78.1%, and 90.1%), owing to the influence of environmental humidity and other factors on the samples not taken into account in the predicted conditions, which lead to the difference in the obtained results. The results prove that the addition of SiO_2_ can prevent the decomposition of PI, whereas Mg(OH)_2_ has little influence.

## 4. Conclusions

The decomposition process of PI is a three-step exothermic reaction under an N_2_ atmosphere. However, the decomposition of PI is a two-step reaction under an O_2_ atmosphere. The first step of the decomposition is unchanged, whereas the peak of the second step transforms into a violent exothermic peak. The results suggests that the first decomposition of PI is irrelevant to O_2_.

The addition of SiO_2_ and Mg(OH)_2_ increase the first step of the decomposition of PI by 4 °C and 7 °C under an N_2_ atmosphere, respectively, but they have no effect on the second and the third step of the decomposition of PI. Under an O_2_ atmosphere, the additives have little effect on the first step of the decomposition of PI and cause the second step of the decomposition temperature of PI to increase by 5 °C.

The kinetic model is proposed to simulate the aging of the three substances at 500 °C for 24 h, and the results of the prediction are consistent with the experimental results. The results also imply that the addition of SiO_2_ can inhibit the aging of PI.

## Figures and Tables

**Table 1:** Experimental materials.

Sample	Specification	Manufacturer
Polyimide (PI)	Thermoplastic, Mw: 50,000–80,000	Nanjing WANQING chemical Glass ware & Instrument Co., Ltd., Nanjing, China
Silica (SiO_2_)	99.5%, 30 nm	
Magnesium hydroxide (Mg(OH)_2_)	AR, 2.86 µm	

AR, which means “Analytical Reagent”, is a purity specification for a chemical reagent.

**Table 2:** Mass loss and peak temperature of the three substances at different stages.

Sample	Ⅰ (450–565 °C)		Ⅱ (565–620 °C)		Ⅲ (620–750 °C)	
	T_p_/°C	∆m/%	T_p_/°C	∆m/%	T_p_/°C	∆m/%
PI	520	21.3	580	48.1	667	29.6
SPI	524	27.6	—	46.7	667	26.1
MPI	527	23.8	583	46.1	667	29.3

T_p_—peak temperature, ∆m—mass loss.

**Table 3:** Multivariate nonlinear fitting results based on the Friedman method.

Kinetic Parameters		PI (A_2_ ^a^ + A_n_^b^ + C_1_ ^c^)	PI/SiO_2_ (A_2_ + A_n_ + Cn ^d^)	PI/Mg(OH)_2_ (A_2_ + A_n_ + F_2_^e^)
Ⅰ	LogA_1_/s^−1^	10.1	6.6	6.1
	E_1_/(kJ/mol)	187.1	134.4	127.6
Ⅱ	LogA_2/_s^−1^	15.1	17.6	15.7
	E_2_/(kJ/mol)	279.6	314.1	290.1
Ⅲ	LogA_3/_s^−1^	13.6	21.5	15.1
	E_3_/(kJ/mol)	294.4	380.4	318.3
	Correlation Coefficient	R = 0.9997	R = 0.9964	R = 0.9998

^a^—2-dim. Avrami–Erofeev; ^b^—n-dim. Avrami–Erofeev; ^c^—1st order with autocatalysis; ^d^—nth order with autocatalysis; ^e^—2nd order.

## Data Availability

Not applicable.
